# Be Happy: Navigating Normative Issues in Behavioral and Well-Being Public Policy

**DOI:** 10.1177/1745691620984395

**Published:** 2021-03-08

**Authors:** Mark Fabian, Jessica Pykett

**Affiliations:** 1 Bennett Institute for Public Policy, University of Cambridge; 2 School of Geography, Earth and Environmental Sciences, University of Birmingham

**Keywords:** behavioral economics, subjective well-being, happiness, public policy, legitimacy

## Abstract

Psychological science is increasingly influencing public policy. Behavioral public policy (BPP) was a milestone in this regard because it influenced many areas of policy in a general way. Well-being public policy (WPP) is emerging as a second domain of psychological science with general applicability. However, advocacy for WPP is criticized on ethical and political grounds. These criticisms are reminiscent of those directed at BPP over the past decade. This déjà vu suggests the need for interdisciplinary work that establishes normative principles for applying psychological science in public policy. We try to distill such principles for WPP from the normative debates over BPP. We argue that the uptake of BPP by governments was a function of its relatively strong normative and epistemic foundations in libertarian paternalism, or *nudging*, for short. We explain why the nudge framework is inappropriate for WPP. We then analyze how *boosts* offer a strict but feasible alternative framework for substantiating the legitimacy of well-being and behavioral policies. We illuminate how some WPPs could be fruitfully promoted as boosts and how they might fall short of the associated criteria.

Psychological science is increasingly influencing public policy. Although it has long played a role in areas such as criminal law, social work, and education, the widespread use of behavioral public policy (BPP) constitutes a milestone. Behavioral insights concerning concepts such as cognitive biases and nonrational decision-making are now applied in a general way across most domains of government. Well-being is emerging as a second domain of psychological science that some people would like to see applied in a similarly general way. However, advocacy for well-being public policy (WPP) has met with opposition in research and political communities. It has been criticized especially for its technocratic attitude and paternalistic proclivities (Davies, 2015; Singh & Alexandrova, 2020). These criticisms are reminiscent of those directed at BPP over the past decade. This déjà vu suggests the need for interdisciplinary work that establishes normative principles for applying psychological science in public policy. These would guide psychologists toward more welcome policy applications of their knowledge and facilitate a smoother translation of psychological science from research communities to government.

In this article, we try to distill such principles for WPP from the normative debates over BPP. We argue that the uptake of BPP by governments was a function of its relatively strong normative and epistemic foundations. The ethical paradigm provided by libertarian paternalism and the causal understanding of behavioral interventions provided by experimental studies made early behavioral interventions robust to many critiques. Nonetheless, substantial critiques remain and have recently led to the development of an alternative normative framework for considering the legitimacy of psychological policies, called *boosts* (Hertwig & Grüne-Yanoff, 2017). The boost paradigm rejects paternalism and the deficit model of citizen psychology by which it is justified. It instead suggests consciously training citizens in psychological insights to enhance their capacities. We argue that WPPs, especially those with the objective of directly improving mental states, cannot be justified under libertarian paternalism. Thus, some ethical heavy lifting is required on the part of WPP advocates if they want to see insights into well-being from psychological science applied in public policy. Fortunately, we argue that the boosts paradigm offers a strict but feasible framework for substantiating the legitimacy not only of BPPs but also of WPPs (and the application of psychological science in public policy more broadly). We illuminate how some WPPs could be fruitfully promoted as boosts and how they might fall short of the associated criteria. We found that the specific policy contexts in which boosts are designed and the populations at which they are targeted are crucial for navigating their normative justification.

## Behavioral Interventions

Behavioral interventions involve the application of insights from behavioral psychology (Kahneman, 2011), notably those concerning cognitive biases, in policymaking (Thaler & Sunstein, 2008). Behavioral scientists have argued that traditional rational-choice theories of economic behavior assume a model of human rationality and utility maximization that is not reflected in empirical reality. As Thaler and Sunstein (2003) put it, “in many domains, people lack clear, stable or well-ordered preferences. What they choose is strongly influenced by details of the context in which they make their choice” (p. 1161). Most early applications concerned economic behavior, such as making savings plans opt out rather than opt in and changing the layout of cafeterias to encourage healthier food choices. Behavioral insights have since been used in a much wider range of applications. In particular, the EAST framework (easy, attractive, social, and timely) developed by the UK’s Behavioural Insights Team is now commonly used as a design principle when thinking about policy implementation (Halpern, 2015). Behavioral interventions involve *nudges*—small changes to the choice architecture confronting a decision-maker that work to remove cognitive biases. This aligns people’s fast-thinking decisions with their slow-thinking preferences.

Nudges can be regarded as problematic because, among other things, they involve government paternalistically manipulating citizens into making certain decisions. In anticipation of these concerns, Thaler and Sunstein (2008) grounded their advocacy of behavioral policy in a broader political philosophy—libertarian paternalism—that tries to reconcile individual freedom of choice and paternalistic means of enhancing public welfare. Libertarian paternalism has seemingly been quite effective at warding off critique given that policy institutions have undertaken to promote the use of behavioral science research globally (Lourenço et al., 2016; Organisation for Economic Co-operation and Development [OECD], 2017, 2019; Van Bavel et al., 2013; World Bank, 2015). We argue that if WPP is to duplicate the policy success of BPP, it will need to be similarly grounded in a sophisticated normative framework. We discuss the key principles and critiques of libertarian paternalism below. We then consider whether WPP could be justified using libertarian paternalism.

## The Normative Foundations of Behavioral Interventions

According to Sunstein (2014), paternalism in BPP is justified because of the clear empirical evidence of “behavioral market failures” to which nudges “are usually the best response, at least when there is no harm to others” (p. 17). Behavioral market failures can be understood as cognitive biases that prevent individuals from making efficient, “rational” economic decisions. They are sometimes called *consumer failures*. Sunstein’s bold assertion is based on normative principles of *effectiveness, autonomy, transparency*, and *welfare* laid out in libertarian paternalism. We analyze these principles below.

### Effectiveness

The claim that nudges are “usually the best response” rests on two of BPP’s key political rationales—nudges “work” and are relatively low cost. They work in the sense that their significant causal impact on behavior is supposedly well established by high-quality experiments, such as randomized controlled trials (RCTs). This evidence exists both for the psychological theories underpinning behavioral interventions and for the interventions themselves. They are low cost because they typically involve merely tinkering with existing policy settings or cosmetic designs, such as using more infographics in communications with citizens, rather than creating whole new policies. As Halpern (2015) pointed out, “Designing policy, and the nuts and bolts of public service, around behavioural insights and empirical methods led to better outcomes, easier services for the public to use, and saved money” (p. 9). We group these appeals to efficiency and “what works” under the heading of *effectiveness* as a justification for behavioral policies.

### Autonomy

BPP is attractive to policymakers in part because of its commitment to promote choice and preserve autonomy. Paternalism as a political critique rests on the harm principle: that it is illegitimate for governments to prevent agents from behaving as they wish unless that behavior causes harm to another person (Conly, 2012). Behavioral interventions skirt this critique by preserving all choices but adjusting the choice architecture to guide citizens toward “better” choices. Critically, the definition of *better* is not provided by the state but by the citizen (see Welfare section below). The choice architecture is adjusted to guide a citizen to that citizen’s own preferred outcome. Behavioral economists have asserted that preference satisfaction is dogged by problems of self-control (Thaler, 2015, p. 86). For instance, one may want to give up smoking but then choose to smoke where cigarettes are easily available and visible. Behavioral interventions such as physically obscuring tobacco product packaging in shops preserve the option to smoke while removing cognitive cues to do so. No choices are prohibited, and no coercion is involved. Libertarian paternalism thus supports autonomy even as it engages in manipulation.

### Transparency

Preempting objections to subliminal state manipulation of citizens, Thaler and Sunstein (2008, p. 244) also identified transparency as one of the guiding principles of libertarian paternalism. Governments adopting policies on the basis of behavioral insights should be willing to defend them publicly. Thaler and Sunstein maintained that nudges are acceptable only when the nudger is open about the intention and mechanism of the nudge. This does not mean that it is necessary to explicitly explain, for instance, that social norms on household energy usage are being framed to encourage proenvironmental behavior according to behavioral insights. Rather, the general motives and methods of government should be publicly known. Thaler and Sunstein drew a distinction between hidden manipulations such as subliminal advertising and visible ones such as graphic warnings of drug side effects. They argued that only the latter are justifiable. This is a relatively weak form of the transparency criterion. A stricter form would be to require the targets of nudges to be explicitly and directly informed as to what nudge is being implemented and why. Nudgers could even be required to explain the psychological mechanisms involved. It seems reasonable to expect policymakers to adhere to stronger forms of transparency where it is easy to do so. For example, ballot bins, which nudge smokers to avoid littering by asking them to vote on some popular culture controversy with their cigarette butts, can easily feature explanations of how they work.

### Welfare

Another central plank in the justification of BPPs is that they offer a novel set of tools for improving people’s welfare however *they* might define it. Sunstein (2014) argued that in the face of a plurality of perspectives on what constitutes a good life, libertarian paternalism enhances welfare by respecting individuals’ own judgments about “how to make their own lives go well” (p. 104). This idea that well-being is whatever they prefer (“desire fulfillment”) is the dominant account of well-being in welfare economics and political theory (Adler, 2019). BPP introduces an important nuance to the preference-satisfaction approach to policymaking by revealing the inconsistency of preferences with and without cognitive bias. It argues that welfare consists in the coolly deliberated, “rational” preferences of individuals. BPP is justified where it can promote this welfare by removing cognitive biases that distort decision-making in the heat of the moment (Lourenço et al., 2016, pp. 9–10). Behavioral interventions are justified because people behave in a “self-destructive” manner in their absence (Loewenstein & Haisley, 2008, p. 211).

### Modesty

A final feature of behavioral policy in practice is that they are typically, although not always, humble in their ambitions. They pursue small targets such as incremental improvements in tax compliance rather than radical policy reforms. The diffusion of the EAST framework across government communications and service delivery is a clear example of this. Few, if any, meaningful policy changes are made; rather, the cosmetics of policy delivery are altered to account for cognitive biases and thereby enhance citizen engagement. This modest quality of behavioral interventions has been seen by some people as a shortcoming (for a discussion, see Chetty, 2015), but it ensures that nudges tend to stay away from especially controversial applications at the boundaries of libertarian paternalism. Curiously, the COVID-19 pandemic has arguably seen these modest aspirations abandoned amid “life and death” rhetoric. Contested evidence about “behavioral fatigue” was seen to play a crucial role in the development of public-health strategy and the justification of liberty-restricting regulation. The value and modesty of BPP has consequently attracted increased public scrutiny (Sibony, 2020).

## The Ethical Boundaries of Behavioral Interventions

We turn now to extant critiques of behavioral policy. Our objective here is twofold. First, we aim to demonstrate that even though it is grounded in the arguably sophisticated normative framework of libertarian paternalism, BPP remains controversial. Second, we aim to illuminate the boundaries of libertarian paternalism and the problematic consequences associated with crossing them. Our intention is not to make an ethical judgment about libertarian paternalism. We try to remain neutral regarding the ethicalness of nudges. Our perspective is that libertarian paternalism has been used successfully to justify the widespread adoption of BPP by governments. This is an empirical observation, not an ethical claim. We suspect that WPP will not be widely adopted by policymakers unless it can be similarly justified. Unfortunately, we argue that libertarian paternalism is a poor fit for WPP. The boost framework fits WPP more easily, and governments (e.g., Germany) that are relatively suspicious of libertarian paternalism seem amenable to boosting. It therefore seems sensible for advocates of WPP to become familiar with boosting and adapt their proposals to fit it. That said, as with libertarian paternalism, we remain neutral regarding the overall ethicalness of boosting.

### Critiques from rationality

We begin with critiques emerging from the way behavioral economics understands rationality and uses it as a normative standard. First, Rizzo and Whitman (2019) argued that nudges rob largely rational individuals of opportunities to autonomously learn about their behavioral biases and institute their own correctives. For example, people on a diet will consciously avoid the confectionary aisle when shopping, empty the house of ice cream, and store cookies out of sight on the top shelf. Choice architecture designed using behavioral insights impedes individuals from becoming more rational over time (Jones et al., 2013). Dworkin (2019) noted how the targeting of policies at cognitive failure and irrationality can end up reinforcing faulty heuristics given that carefully designed choice architectures allow bad reasoning to lead to good outcomes. Nudges can thus be opposed to the democratic ideal of acting in accordance with and in *conscious recognition of* reasons.

Second, White (2017) raised the concern that if cognitive biases, dysfunctions, and limitations are as pervasive as behavioral economists argue, then revealed preferences constitute a questionable foundation for welfare analysis. The legitimacy of nudges hinges on the ability to identify underlying “rational” preferences that would be revealed in choices if only biases were removed. The abundance of biases identified to date suggests that the identification of these “true” preferences might be impossible, if they exist at all.

Both critiques point to a third, metaissue: the need for some theory of subject formation to ground the nudge paradigm (Jones et al., 2013; Pykett, 2012; Rebonato, 2012). What we mean by a theory of subject formation here is an account of where preferences come from. This is a long-standing black box in economics created by the neoclassical assumption of exogenous preferences. Preferences are of course *endogenous*. They are shaped by the agent’s social context (Bowles, 1998; Hoff & Stiglitz, 2016) and constructed and developed by the agent over time in response to new information. The psychological sciences similarly host a myriad of divergent perspectives on subject formation, from theories of evolutionary neurobiology to psychosocial accounts. These contextual and cognitive issues can easily be political. For example, social scientists have emphasized that revealed preferences might emerge from a context that is problematic, such as conformism to oppressive regimes. Why should such preferences be taken as an appropriate standard of welfare? BPP needs an account, potentially on a nudge-by-nudge basis, of the introspective process by which true preferences come about and can be identified by policymakers (Dworkin, 2019; Sunstein, 2017; Wilkinson, 2013).

### Critiques from technocracy

We turn now to a collection of critiques that can be grouped under the heading of technocracy. Liberal political theory has always been concerned about the question of who rules the rulers. This is obviously a concern in behavioral interventions in which policymakers largely assume citizens’ true preferences. What is to stop policymakers passing off their own preferences as those of citizens? In a recent account of the behavioral insights movement in the Dutch government, Feitsma (2018) considered whether the proliferation of behavioral insights teams, behavioral experts, and choice architects in government can be criticized on the basis of a renewed *technocracy* and *psychocracy.* Here, bureaucrats and decision-makers are guided by psychological expertise rather than public dialogue. Such concerns are taken seriously by people promoting nudge policies. Sunstein (2014) asked, “Who will monitor the choice architects, or create a choice architecture for them?” (p. 16). Likewise, the UK’s Behavioural Insight Team published the behavioral government report (Hallsworth et al., 2018) to demonstrate how to work around the fact that “elected and unelected government officials are themselves influenced by the same heuristics and biases that they try to address in others” (p. 7).

A second line of critique attacks the way the evidence-based policy paradigm presents properly normative and political questions as technical and therefore to be resolved by experts rather than the public (Sanderson, 2003; Sullivan, 2011). Far from being nonideological, the way the rationales of policy effectiveness, cost efficiency, and “what works” are operationalized constitutes a normative agenda (Triantafillou, 2013). By reducing the welfare argument to a technical debate about the existence of systematic, empirically evidenced cognitive biases that universally plague decision-making, the need for normative justifications at all is brushed away (Rebonato, 2012, p. 394).

Third, critics have questioned the degree to which BPP focuses responsibility for public welfare on individuals. Many interventions target and potentially demonize certain behaviors that may not be caused so much by cognitive biases as by objective social conditions and life circumstances such as poverty. In some cases of poor welfare outcomes, causation might ultimately lie in poor policy settings, such as urban planning that leads to sprawl, limited public transport, and food deserts. In this case, policy effectiveness should not be evaluated on the basis of behavioral change in narrow experimental conditions. Instead, evaluation should consider whether policy contributes to the realization of just conditions (Prainsack & Buyx, 2014) and the rectification of the deep causes of the social problems to be tackled (Quigley & Farrell, 2019).

### Critiques from deliberation

Riley (2017) raised a concern about the epistemic justice of nudges that pulls in themes from each of the above lines of critique. Epistemic injustice affects a citizen in his or her capacity as a knower. Nudges arguably involve epistemic injustice because they work through the stimulation of “non-deliberative cognition” (Riley, 2017, p. 600). The citizen’s ability to exercise his or her reasoning and judgment are bypassed in nudges on the grounds that these faculties are plagued with bias. Riley was concerned that the cumulative project of nudging across the gamut of public policy could restrict people’s willingness and ability to reasonably deliberate “amongst other recognised epistemic and cultural peers” (Riley, 2017, p. 604). This would limit situations in which they can be involved in setting agendas and deliberatively arriving at the public goals of public policy. The proliferation of nudges could pull an increasing number of policy domains away from citizens and their capacity to act in the future, giving responsibility instead to technocrats.

### Critiques of causal inference

A final cluster of critiques concerns the evidence and theory that underpins behavioral science and nudges. Studies continue to emerge in which the existence and/or severity of many of the behavioral biases invoked in BPP are questioned (Gal & Rucker, 2018; Yechiam, 2019). Other studies have failed to replicate the effects of specific behavioral interventions (Jachimowicz et al., 2019; Shadel et al., 2019). Indeed, in a recent meta-analysis of a large range of nudges, Hummel and Maedche (2019) found that only 62% were statistically significant. These results undermine claims to effectiveness in BPP.

More broadly, Grüne-Yanoff (2017) argued, somewhat controversially, that far from providing explanations of decision-making, “behavioral economists typically engage in developing *as-if* models: namely, models that fit the behavioural phenomena, but [make] no (legitimate) claim to the underlying psychological mechanisms that brought about this behaviour” (p. 67; see also Berg & Gigerenzer, 2010). In the place of explanation, behavioral science offers instead correctives and qualifications to existing models and new constructs that correlate with experimental evidence. In so doing, psychological perspectives have been misrepresented as the science of irrational decision-making (Gigerenzer, 2018). Grüne-Yanoff (2016, p. 480) further argued that behavioral economics cannot offer robust and effective evidence to justify nudge policies that will work in the long term or in general equilibrium, and so governments should not rush to implement BPP on the basis of current knowledge. Along with the failures to replicate outlined above, these critiques call into question claims from nudge advocates about how settled behavioral science is.

This critique of the mechanistic ambiguity of nudges is related to a more general critique of RCTs concerning their external validity (Deaton & Cartwright, 2018). Although RCTs can provide tight causal identification (internal validity), their findings are typically hard to generalize to other circumstances (external validity). Behavioral interventions may be more robust to this charge than other areas of policy because they claim knowledge of near-universal characteristics of human decision-making. Regardless, there may be a need to combine RCTs with other methods of evaluation to understand the effects and effectiveness of behavioral policies in a rich, contextual way.

## From Behavioral Insights to Well-Being

These four lines of critique suggest that psychological interventions can be controversial even if they conform to the normative requirements of effectiveness, autonomy, transparency, and welfare proposed by libertarian paternalism. There is currently a lack of evidence to empirically assess the severity of these issues across behavioral policymaking. Some research exists on the public acceptability of hypothetical nudges in different international contexts that underlines the importance of trust in public institutions (Sunstein et al., 2019). However, there is a dearth of research on the long-term and cumulative effects of behavioral public policies and few accounts of the lived experiences of being involved in behavioral interventions (for an exception, see Cohn & Lynch, 2017). Nudging, then, is arguably popular but at least somewhat controversial, and caution is required if behavioral interventions are to be spread more widely and scaled up.

We turn now to applying the insights gained from our analysis of the foundations and limits of libertarian paternalism to nascent WPP. We take WPP to include two items. The first is “happiness interventions,” which are aimed at improving the *mental states*, notably emotional health, of specific populations, such as in schools, workplaces, or place-based communities. *Mental states* include feelings of meaning, purpose, accomplishment, engagement, and other states sometimes associated with psychological well-being, not just happiness. The second is *government well-being budgeting*, which prioritizes policy spending to improve the aggregate life satisfaction of the population (Frijters et al., 2020). We distinguish such WPP from the promotion of “economies of well-being,” such as in New Zealand, Iceland, Scotland, or Wales. The latter is a broader and more explicitly political agenda that uses social indicators and composite quality-of-life metrics to orient public policy away from economic growth and toward creating the objective conditions for inclusive, fairly distributed, and sustainable prosperity. We argue that happiness interventions and well-being budgeting transgress the principles of effectiveness and autonomy and that their adherence to the principle of welfare is questionable. Initial indicators of transparency are more encouraging.

### Effectiveness

Advocacy for BPP was founded on experimental studies of both psychological mechanisms such as present bias and specific policy interventions based on those mechanisms, such as default schemes. Experimental evaluation in WPP is increasingly strong on psychological mechanisms but remains weak on policy applications (Clark et al., 2018). To mimic the success sequence of BPP, WPP will also need to demonstrate cost-effectiveness. Lordan and McGuire’s (2018) evaluation of the UK’s Healthy Minds curriculum, aimed at improving psychological resilience and improving health and behavior, provides a template for how to do this.

Some people have proposed WPPs are particularly weak on the “what works” front. Well-being budgeting, for example, is heavily reliant on correlation analysis that produces inconclusive results. It might be feasible at a micro level in which two policies suited to a niche area can be compared robustly on the basis of experimental evidence. For example, if a government decided to introduce mood-management training in schools to promote subjective well-being (SWB), it could compare the cost-effectiveness of the Healthy Minds and ENHANCE programs (Heintzelman et al., 2020). However, our current understanding of SWB at the macro level, at which experiments are challenging, is limited. For example, Clark et al.’s (2018) regression analysis of the “origins of happiness” has an *R*^2^ of .14 (i.e., the regression explains only 14% of the variation in life satisfaction). Their regression for childhood well-being has an *R*^2^ of only .03. Using such unclear results to decide spending priorities is not “modest” and invites unintended consequences and misplaced resources.

Oishi et al.’s (2018) argument for designing taxation policy according to life-satisfaction outcomes illustrates these risks. Using time-series regression modeling, they showed that periods of relatively high progressive taxation were associated with relatively large increases in the life satisfaction of the poor and only relatively small decreases in the satisfaction of the rich. Oishi et al. argued that taxation should therefore be more progressive because this will increase total SWB. Leaving aside the contentious politics of inequality and taxation, Oishi et al.’s methods did not establish that progressive taxation caused the discrepancies in life satisfaction. Note that they did not assess whether it was higher social security spending during periods of high taxation that was the real causal element in the higher rates of satisfaction among the poor. If it is social security that increases life satisfaction, then increasing taxes will have little direct effect. Meanwhile, the taxation could depress consumption and increase unemployment for poor people, reducing their life satisfaction. Establishing causation in a manner suitable for policy work will likely require psychologists to become more familiar with the nonexperimental methods for establishing causation developed by other social sciences, notably economics, as advocated recently by Grosz et al. (2020).

A final effectiveness issue for WPP is residual concerns around the measurement of psychological well-being, especially SWB. Many early criticisms around question order, effects of day of the week, social-desirability bias, and other peculiarities of subjective response (Bertrand & Mullainathan, 2001) have been largely addressed through improved survey design and question wording (National Research Council, 2013; OECD, 2013). However, concerns remain about the noncomprehensiveness of single-item SWB measures (e.g., life-satisfaction scales), how people interpret questions, and scale norming (Benjamin et al., 2020). Scale norming seems a particularly pernicious issue in a policy context because it biases statistics in a way that undermines cost-effectiveness analysis (Adler, 2013). In scale norming, the qualitative meaning of the points on a respondent’s scale changes between waves of a survey (Fleurbaey & Blanchet, 2013). It is called *response shift* in the quality-of-life literature (Ubel et al., 2010). In a meta-analysis of response-shift studies, Schwarz et al. (2006) found consistent evidence for its existence, although effect sizes were small to moderate.

There are other measures of psychological well-being besides SWB, notably those associated with so-called eudaimonic perspectives on well-being (Marsh et al., 2020; Ryff, 1989). Although these rely on self-reports as much as SWB metrics do, they aim at objective constructs such as basic psychological needs. However, these measures tend to be highly multidimensional, which makes them unsuitable for use in cost-benefit analysis (Fleurbaey & Blanchet, 2013). Furthermore, scholars in the subjective well-being tradition have argued that these measures, if valid, have not been applied extensively at sociological scale and are therefore too immature to be used in public policy at this stage (National Research Council, 2013).

### Welfare

The way psychologists define well-being can be problematic in a policy context. It is important to understand that well-being simpliciter is not an ateological concept. It must be defined before it can be measured and is quite nebulous (Alexandrova, 2017). This definitional process is often obscured in disciplinary research silos that each have their own midlevel theory of well-being. Well-being is a value-laden concept, meaning that its definition involves making a value judgment, and so what definition is ultimately settled on has normative implications (Prinzing, 2020; Tiberius & Hall, 2010). For example, if well-being is defined as “happy” mental states, then government cost-benefit analyses will seek to make citizens happier even if happiness is not citizens’ preference. This can lead to (soft) authoritarianism, something political ethicists spend much time theorizing about but is rarely salient in psychological research. It is because of this value-laden nature that philosophers treat well-being as synonymous with the *prudential good* (Bishop, 2015), that is, what is “good for” individuals. This makes the normative associations of the concept apparent. Psychologists must be sensitive to how the normative implications of how well-being is defined shift in the transition from laboratory to policymaking.

Much WPP advocated by psychologists employs a mental state account of well-being. For example, Diener et al. (2009) advocated for policies to improve SWB, which they defined as “good mental states,” including satisfaction with life and a preponderance of positive affect over negative affect. SWB might be a suitable definition of well-being in the context of psychological science, but such mental-state definitions have long been anathema to philosophers and widely considered inappropriate for policy. We review the preeminent critiques below.

The first concern with defining well-being as a mental state in a policy context is the already mentioned potential for ignoring citizen preferences. For example, Clark et al. (2018) argued, in the context of migration policy, that “the key to happiness is that the circle of sympathy is extended as widely as possible” (p. 122). Governments could use such logic to justify increasing migration because it makes everyone happier even if citizens express a preference for lower immigration. Benjamin et al. (2012) demonstrated that happiness and preference do not always coincide. They presented survey respondents with several paired choices. They asked first, “Which one would make you happier?” and then “Which one would you choose?” They found that although happiness and choice coincided on average in 83% of cases, on some questions, coincidence was below 50%. Western political philosophy has long been concerned about the coercive power of the state and averse to normative paradigms that allow the contravention of citizens’ wishes. Well-being is consequently often defined as preference satisfaction in the policy domain (Adler, 2019). WPP advocates should be cautious about suggesting that policy should override “dumb” or “bad” preferences that undermine citizens’ SWB. This might be reasonable in a therapeutic context, but it is a redline in policy.

The second concern with mental-state definitions of welfare is what economists have called *adaptive preferences* (Sen, 1999). It is well established that people’s life satisfaction can acclimatize to their circumstances. Graham (2011) coined the “happy peasant” and “frustrated achiever” concepts to describe the phenomenon of impoverished individuals who report high levels of life satisfaction and objectively well-off individuals who nonetheless report relatively low levels of satisfaction. Life satisfaction has also been found to recover, often completely, to a set-point level over time following many shocks, including marriage, divorce, income growth, and even spinal injury (Sheldon & Lucas, 2014). Adaptation has two arguably perverse consequence for WPP. First, it means that government will focus policy resources (effort, attention, money) on frustrated achievers rather than happy peasants even though the peasants are objectively worse off. From the point of view of distributional justice, this seems wrongheaded. Second, if promoting adaptation is cheap, cost-effectiveness will often demand that governments help citizens adapt to adverse conditions rather than ameliorating those conditions. For example, it may be cheaper to be provide therapy to someone depressed about their spinal injury than to fix the spine. In such circumstances, adaptation seems to let government off from its responsibilities.

The third problem with mental-state policy is its anesthetizing effect on political sentiment. Critical theorists have stressed that bad moods and dissatisfaction are important catalysts for justifiable political activism (Davies, 2015). When governments focus on improving such negative mental states by treating the feelings rather than their causes, they dissipate the political energy required to drive deep reforms. For example, policy targeting the mental health of homeless people might inadvertently undermine feelings of despair, anger, frustration, outrage, and agency that would otherwise lead to an autonomous and effective campaign for affordable housing. Acknowledging this, “Housing First” homelessness reduction programs, which do not make housing conditional on substance abstinence, are more successful in preventing long-term homelessness and ill health (Baxter et al., 2019).

Mental-state policy is particularly perverse when it results in victim blaming. Friedli and Stearn (2015) gave the example of the U.K. government making welfare payments conditional on attending positive psychology sessions. This shifts responsibility for unemployment from the government’s procyclical austerity policies to citizens’ lack of a “positive attitude.” WPP needs to be mindful that it does not further burden already disempowered people with the need to feel happy and enthusiastic despite their situation.

Concerns around adaptation and political disempowerment are one reason why many areas of policy, such as the millennium development goals, are dominated by the *capabilities* definition of well-being. Capabilities are the options available to citizens in terms of who they can be and what they can do (Nussbaum, 2000). For example, a homosexual person in a country with same-sex marriage rights has more capabilities than an identical individual in a country without such rights and therefore has higher well-being. The focus of policy in the capabilities framework is on increasing the opportunities available to citizens through education, health, income growth, political enfranchisement, and capacity building. Citizens choose how to leverage these opportunities to satisfy their preferences. The capabilities approach ensures governments improve objective circumstances rather than how people feel about those circumstances. The boosts framework that we outline below resembles capabilities in that it promotes citizens’ psychological capacities. This allows for mental states to be promoted *indirectly*—the policy objective is greater capability, but a by-product could be higher SWB.

Our discussion of welfare reveals two further points. First, well-being can be defined in a multitude of different and often incompatible ways. Subjective well-being or happiness in the sense of self-assessed affect and life satisfaction is but one (mental state) account. We are concerned about the tendency in WPP advocacy to define well-being as a mental state and to then argue, implicitly or explicitly, that competing approaches are not about well-being. Income and prices, for example, may be a poor measure of SWB, but they are an effective measure of preference satisfaction (Angner, 2009). Second, what well-being is and how a government should go about promoting it are two separate questions. Even if there were broad consensus that SWB is simply “well-being,” it might nonetheless be inappropriate for government to promote it in certain ways because of paternalism, adaptive preferences, and other issues discussed above.

### Autonomy

Some advocates of WPP have argued that SWB is more respectful of autonomy than existing preference-satisfaction and capabilities approaches. For example, Clark et al. (2018) wrote that life satisfaction “is democratic—it allows individuals to assess their lives on the basis of whatever *they* consider important to themselves” (p. 4). Likewise, Frijters et al. (2020) wrote that a life-satisfaction scale “takes individuals seriously as political agents and sets them at the top of the judgment tree” (p. 16).

This claim is questionable. Respondents to SWB survey questions are never asked why they hold a level of life satisfaction. Instead, their numerical responses are placed on the left side of a regression and variables chosen *by the researchers* are used to determine what causes respondents’ levels of satisfaction. This is a sensible research practice. Asking respondents what determines their satisfaction directly could contaminate responses with present, social-desirability, and other biases (National Research Council, 2013). Unfortunately, this practice is problematic in a policy context in which identifying and explaining subjective biases is central to the process of policy deliberation. The practice uses cognitive failings to justify bypassing the voice of citizens, cutting them out of deliberation, and giving preeminence to experts in decision-making. This was precisely the critique leveled at nudging, so WPP seems to have little advantage here. Stutzer (2019) pointed out that people derive life satisfaction from liberal-democratic processes themselves independently from the outcome of those processes. Thus, any well-being proposals derived from expert analysis of life-satisfaction data should be taken to the public for deliberating and vetting before being implemented as policy. This two-step approach would avoid contaminating life-satisfaction responses while preserving citizen autonomy.

### Transparency

Transparency is a major issue for nudges because of their potential to be subliminal in practice. For example, Hansen and Jespersen (2013) noted that many nudges, such as offering only smaller plates in cafeterias to reduce food waste, do not engage reflective thinking. Nudging for happiness would have similar issues, but much WPP is not remotely subliminal. Furthermore, early indications from jurisdictions in which WPP is prominent, such as Bhutan, suggest that government tends to be explicit when its policy objective is well-being. Indeed, governments tend to shift to WPP amid much fanfare, as in New Zealand’s well-being budget. One issue for transparency that may arise as circumstances develop is whether a government is explicit about the kind of well-being it is promoting. For example, well-being in the capabilities framework is markedly different from well-being in terms of mental states.

## Boosts: A Way Forward for WPP

WPP would be hard to justify under libertarian paternalism at this time because of its limited effectiveness credentials and tendency to transgress the principles of welfare and autonomy. Although these may not be relevant in research and therapeutic contexts, they become critical in the policy domain in which state power and democratic legitimacy are salient considerations. An alternate framework that could be helpful to guide WPP to legitimate applications is the boosts paradigm (Hertwig & Grüne-Yanoff, 2017). In contrast to nudging’s emphasis on technocratically guided choices, boosts focus on improving citizens’ capacity for self-guidance. Broad categories of boosts include skills training, explicit persuasion and information representation, and assistance for subjects to inculcate habits or routines based on psychological science that promote welfare through, for example, financial literacy, risk assessment, health-promoting choices, informed decisions, and “effective self-regulation” (Hertwig & Grüne-Yanoff, 2017, p. 982).

Boosting builds on Gigerenzer’s critiques of behavioral economics and promotes an alternative account of psychology: *ecological rationality* (Gigerenzer, 2018; Gigerenzer & Brighton, 2009), in which people’s nonrationality is taken as an adaptive capacity to be valued under conditions of uncertainty rather than a cognitive error to be corrected (Hertwig et al., 2019). Relatedly, whereas behaviorism views the mind as profoundly irrational, with biases as the rule rather than the exception, boosting draws on a different body of literature in psychology that emphasizes the immense human capacity to learn and develop competence. Gigerenzer (2018) argued that instead of paternalistic interventions such as nudging to correct biases, public policies should aim “to hone the skills of the general public in dealing with risks and making decisions” (p. 311).

Gigerenzer (2018) considered the German chancellery’s citizen-centered approach to behavioral insights and policy testing exemplary in this regard. Yet the policy application of boosts in Germany is somewhat underdeveloped. Since 2015, the federal government there has favored information framing and educational campaigns. Examples include hospital hygiene, vaccination, and consumer protection (German Federal Government, 2020). These initiatives are based on narrow forms of customer insight or citizen consultation. There is therefore scope for elaborating the psychological and normative basis of boosting and identifying potential applications in WPP.

Boosts differ from nudges in several respects. First, they are educative and sustainable over the long term. We call this the principle of *capacity.* Boosts enhance citizens’ understanding of psychological processes in ways that they can leverage to benefit their welfare. Boosts therefore expand people’s capabilities—the things they can be and can do. This means that boosts avoid issues of adaptive preferences and keep government focused on objective circumstances, not feelings. In this vein, the Netherlands government now subjects public policies to a capacity to act test that requires legislators to consider whether policies are based on “realistic assumptions about people’s mental resilience” (Netherlands Scientific Council for Government Policy, 2019). Like nudging, this recognizes limits to rationality but is more boosting in nature because it promotes action to increase mental capacity and self-determination in the long term.

Second, boosts avoid assuming a deficit model of cognition and behavior. They do not conceptualize psychological phenomena such as cognitive biases as failings but instead see them as adaptations whose utility varies across contexts. Boosts focus on helping recipients better understand how their psychology manifests in different spaces. We call this the principle of *empowerment*. It mitigates the potential for victim blaming in psychologically informed policy, deflects accusations of dumb or bad preferences, and resists efforts by the powerful at subordinating the weak on the grounds of their deficient psychology.

Ideally, boosts should be empowering in the context of their implementation, transferable between contexts, and durable over the longer term. There is a significant difference between, say, promoting decision-making capacity as a learned mental skill and empowering people to improve their well-being by autonomously transforming aspects of their social context. For example, WPP for the homeless may be of marginal usefulness if it does not assimilate the powerlessness of their situation.

Finally, boosts require not only transparency but also active and conscious cooperation from persons being boosted (Hertwig & Grüne-Yanoff, 2017, p. 982). We call this the principle of *participation.* It protects against paternalism and manipulation and engages citizens in a way that fosters deliberation and democratic oversight of policy choices and outcomes. An important dimension of participation is that it connotes a collective activity, challenging the methodological individualism found in some strands of psychology.

We turn now to some examples of how these principles might be navigated in WPP. We discussed earlier the problematic use of positive psychology as part of conditionality for receiving welfare payments. One could mistakenly see such programs as boosts because they train welfare recipients in psychological skills. This satisfies the capacity principle. However, such programs transgress the empowerment and participation principles. On empowerment, they assume that psychological deficits (i.e., a negative attitude and learned helplessness) lie behind welfare recipients’ inability to find employment. Recipients’ attitudes may instead be a normal response to overwhelming environmental conditions of poverty, family breakdown, precarious work contracts, and limited social capital. On participation, because welfare recipients’ livelihoods depend on attending these conditional programs, they act under duress, and their perspectives on the complex barriers to their employment are rarely given voice.

School-based emotional-management programs such as Healthy Minds offer an instructive contrast. Such programs develop psychology skills, satisfying the capacity principle. Education is a domain in which paternalism and consent are generally considered less problematic. If such programs are universal rather than targeted at underperforming or emotionally volatile students, they may satisfy the empowerment principle and pay due regard to social context. Instructors would have to be sensitive to how students whose early childhood experiences were detrimental to their learned capacity for emotional regulation may react to the curriculum. However, if new skills are framed in terms of wisdom rather than treatment, they could have an empowering rather than demoralizing effect. Note that Hertwig and Grüne-Yanoff (2017, p. 982) differentiated between boosts and schooling. However, Healthy Minds satisfies their criteria for this distinction. In particular, it builds motivational and decisional competencies on the basis of psychological science. It is also quick and delivered “just in time” for the onset of late adolescence, a volatile period for well-being.

Finally, what we call *social boosts* have the potential to reconfigure WPP from an expert-led agenda dominated by economists to a more interdisciplinary, participatory, and context-sensitive approach to public policy design. Social boosts are investments in community infrastructure, both built and social, such as meeting places, neighborhood design, and green and blue spaces and clubs. Recent examples include the Men in Sheds program to tackle social isolation, community-gardening initiatives, and active travel interventions. Social boosts are inherently participatory and focused on enhancing community capacity.

In an evidence review, Bagnall et al. (2018) found that public interventions aimed at reshaping social relationships through physical environments can improve both individual and community health and well-being, social relations, social trust, and social capital. Such outcome measures draw on methodological and epistemological perspectives from community psychology. They emphasize that people are embedded in groups and that individual well-being is therefore inextricably tied to group well-being. This approach diverges from the individualistic paradigm associated with SWB. It defines community well-being as “the combination of social, economic, environmental, cultural, and political conditions identified by individuals and their communities as essential for them to flourish and fulfil their potential” (Wiseman & Brasher, 2008, p. 358). Examples include asset-based community development in health care promotion and schemes to enhance collective physical, voluntary, or civic activity. These were found to enhance people’s skills and confidence to act independently in the future, meeting the empowerment principle (Bagnall et al., 2018).

## Conclusion

In this article, we drew lessons from the history of behavioral policy for the nascent push to inject psychological insights concerning well-being into public policy. We reviewed the four normative principles underpinning the relatively successful reception of behavioral science among policymakers and citizens: effectiveness, autonomy, transparency, and welfare. We then explained some of the major critiques of these principles of libertarian paternalism. These critiques demonstrate the normative complexity of transferring psychological insights from the laboratory to policymaking. Many of these critiques apply just as readily to proposed well-being public policies as they do to behavioral policy. We argued that WPP that targets mental states is especially prone to transgressing the normative principles of libertarian paternalism. We offered the boosts paradigm as an alternate framework for considering the normative legitimacy of well-being public policies. We distinguished three normative principles that characterize boosts: capacity, empowerment, and participation. Adherence to these principles allows well-being boosts to be justified within the capabilities definition of well-being, which is one of the more widespread conceptualizations of the term in the policy space. These arguments are summarized in Table 1, which presents the pros and cons of libertarian paternalism and boosts for BPP and WPP, respectively.

**Table 1.** Pros and Cons of Normative Paradigms for Applying Psychological Science in Public Policy

Normative paradigm	BPP		WPP	
	Pros	Cons	Pros	Cons
Libertarian paternalism				
*Effectiveness*: Relatively cheap to bring about statistically significant effects with well-understood causation *Autonomy*: All choices still available; no coercion *Transparency*: Policymaker must at least be open about the intention and mechanism of the psychological intervention *Welfare*: The realization of the recipient’s own preferences is the policy objective	Promoted the uptake of BPPs by policymakers by providing them with a sophisticated and relatively firm normative foundation Straightforward set of criteria Restrictive, which is appropriate given the potential for manipulation, but open enough to admit many applications of psychological science in policymaking Modest: not a major departure from prevailing principles of policymaking such as the welfare principle and “what works”	Impedes recipients from becoming more “rational” and psychologically sophisticated over time Does not respect recipients’ reasons for their behavior Limited recognition of social context and subject formation Technocratic/psychocratic Does not involve citizens in policymaking Epistemic injustice Dependent on very strong scientific evidence for legitimacy	An already existing, arguably well-developed, and successful normative paradigm that could be used to legitimate some subset of WPPs	Technocratic/psychocratic Welfare principle is almost impossible to respect when the objective is mental states rather than preference satisfaction or capabilities Hard to fit many WPPs into libertarian paternalism because WPPs are often not behavioral in nature and do not involve correcting cognitive biases Scientific evidence is not strong enough to meet effectiveness criteria for most WPPs at this time
Boosting				
*Capacity*: Educative and sustainable over the long term *Empowerment*: Helps recipients better understand their psychology and how to leverage it *Participation*: Conscious cooperation and informed consent	Avoids many cons for BPP listed above, for example: Empowerment and participation mitigate technocracy and involve citizens in policymaking Capacity helps citizens become more (ecologically) rational over time	Time-consuming and involved process Expensive Stricter framework that admits fewer policies (e.g., many nudges ruled out)	Allows for many WPPs that would be ruled out by welfare principle because participation principle ensures consent and deliberation rather than manipulation of preferences Encourages policymakers to involve citizens in the policymaking process	Stricter framework that rules out many interventions Requires large-scale participatory and/or deliberative processes for high-level policy applications such as cost-benefit analysis protocols and national statistics Expensive to apply

In the final part of the article, we discussed some examples of WPP boosts, including psychological skills training in the context of unemployment policies and school curricula and social boosts for community well-being. We illustrated policy contexts in which these boosts could be useful and legitimate and might be normatively suspect according to the three principles. We hope that our analysis is helpful to individuals and organizations interested in pursuing policy applications based on behavioral insights and psychological perspectives on well-being.

## Transparency

*Action Editor:* Joshua Hicks

*Editor:* Laura A. King
